# Long-covid cognitive impairment: Cognitive assessment and apolipoprotein E (APOE) genotyping correlation in a Brazilian cohort

**DOI:** 10.3389/fpsyt.2022.947583

**Published:** 2022-08-10

**Authors:** José Wagner Leonel Tavares-Júnior, Danilo Nunes Oliveira, Jean Breno Silveira da Silva, Werbety Lucas Queiroz Feitosa, Artur Victor Menezes Sousa, Letícia Chaves Vieira Cunha, Safira de Brito Gaspar, Carmem Meyve Pereira Gomes, Laís Lacerda Brasil de Oliveira, Caroline Aquino Moreira-Nunes, Raquel Carvalho Montenegro, Manoel Alves Sobreira-Neto, Pedro Braga-Neto

**Affiliations:** ^1^Neurology Section, Department of Clinical Medicine, Faculty of Medicine, Federal University of Ceará (UFC), Fortaleza, CE, Brazil; ^2^Drug Research and Development Center (NPDM), Pharmacogenetics Laboratory, Federal University of Ceará (UFC), Fortaleza, CE, Brazil; ^3^Center of Health Sciences, State University of Ceará, Fortaleza, CE, Brazil

**Keywords:** COVID-19, cognitive impairment, SARS-CoV-2 infection, dementia, risk factor

## Abstract

**Introduction:**

Few studies have objectively evaluated cognitive deficits after the acute phase of COVID-19 disease. Moreover, the role of apolipoprotein E (APOE) genotypes in cognitive decline in patients with COVID-19 has not been evaluated yet.

**Methods:**

This cross-sectional study was conducted in confirmed cases of COVID-19 patients with neurological symptoms that persisted for more than 3 months from the onset. We determined APOE genotypes.

**Results:**

The final sample consisted of 141 patients. The most frequent APOE genotype was E3/E3 (*N* = 95; 67.3%). In total, 93 patients (65.9%) had memory impairment symptoms as the main complaint, objectively confirmed through screening tests in 25 patients (17.7%). Patients with cognitive impairment had a lower frequency of anosmia than the normal and subjective cognitive decline (SCD) groups (*p* = 0.005). In addition, depression was recurrent in the cognitive impairment group and the SCD group (*p* = 0.046). Cognitive impairment was significantly more frequent in hospitalized patients and those with a lower education level. Cognitive status was not associated with APOE genotypes.

**Discussion:**

Hospitalized patients had more severe infection with a greater possibility of systemic complications, greater inflammatory response, and prolonged hospitalization, which could impact cognitive performance. Cognitive impairment in patients with COVID-19 does not necessarily involve specific APOE polymorphisms. However, psychiatric disorders may also be responsible for cognitive complaints. Cognitive complaints are frequent in patients with COVID-19, even after the acute phase of the disease and in mild cases. Hospitalized participants and depressed patients may have a higher risk of cognitive impairment. APOE genotypes or haplotypes may not significantly play a role in COVID-19 cognitive impairment.

## Introduction

The COVID-19 outbreak started in Wuhan, China and was declared a pandemic by the World Health Organization (WHO) on 11 March 2020, with high infection and mortality levels worldwide.^1^ COVID-19 has a wide range of clinical manifestations, such as neurological manifestations (1, 2). In a study conducted in Wuhan, 36.4% of the patients had some neurological manifestation, with central or peripheral neurological involvement, such as dizziness, headache, altered level of consciousness, stroke, ataxia, and epilepsy (3).

Aside from general neurological manifestations, cognitive impairment was evaluated in different COVID-19 phases. A Chinese study evaluated the cognition of 29 patients with COVID-19, correlating cognitive complaints to high C-reactive protein (CRP) levels during the disease's acute phase (4). Another study evaluated cognitive impairment in outpatients using the Montreal Cognitive Assessment (MoCA), finding cognitive impairment in patients with mild symptomatic COVID-19 after 12 weeks of COVID-19 onset (5). Moreover, different cognitive presentations have been described in acute patients, such as encephalopathy associated with severe conditions and akinetic mutism associated with frontal hypometabolism in brain fluorodeoxyglucose (FDG)-PET (6, 7).

More severe COVID-19 manifestations in patients have been correlated with the APOE-4 allele of the apolipoprotein E (APOE) gene (8). This association is significant since the same allele confers a higher risk of sporadic Alzheimer's disease (AD) (9). Furthermore, a previous study showed that single nucleotide polymorphisms (SNPs) rs429358 and rs7412 of the APOE gene are associated with ischemic cerebral infarction, which is essential given the contribution of cerebrovascular diseases in the pathophysiology of many dementia cases (10, 11). As far as we know, no publications have evaluated cognitive manifestations after COVID-19 and correlated them with APOE polymorphisms. Similarly, we observed a limited number of studies in the literature evaluating cognitive manifestations in patients after the COVID-19 acute phase.

This study aimed to determine the relationship between COVID-19 and cognitive impairment and APOE gene polymorphisms in an outpatient public university hospital in Northeast Brazil.

## Methods

### Patients and clinical assessment

This cross-sectional study was conducted with COVID-19 outpatients at the Walter Cantídio University Hospital in Fortaleza, Northeast Brazil. Patients were recruited from July to August 2020 from an ongoing prospective longitudinal study by our research group.

We included patients with a diagnosis of COVID-19 confirmed in the past 12 months by nasal swab reverse transcription (RT)-polymerase chain reaction (PCR) or serological test, with any neurological symptom that persisted for more than 3 months from the onset. We excluded patients who did not undergo confirmatory testing for COVID-19 and those without neurological complaints (e.g., headaches, cognitive complaints, and others). Evaluations were performed in the neurology outpatient clinic of the Walter Cantídio University Hospital of the Federal University of Ceará, Brazil.

Patients were clinically evaluated by two independent neurologists (JWLTJ and DNO). The same clinical evaluation and identification form was applied to all patients. Age, gender, schooling, initial neurological symptoms, hospitalization, COVID-19 test type, complementary tests, comorbidities, alcohol abuse, and tobacco history were questioned. Moreover, the Medical Research Council (MRC) dyspnea scale was applied to assess dyspnea before and after COVID-19 (12).

### Cognitive assessment

Participants were submitted to Addenbrooke's Cognitive Examination-Revised (ACE-R), the Mini-Mental State Examination (MMSE), and the Clinical Dementia Rating (CDR). Pfeffer's instrumental activities of the daily living scale were applied to assess functionality, and the Geriatric Depression Scale (GDS) was applied to assess mood, or the Beck Inventory, depending on the age of the patient (13–18). Furthermore, the Prospective and Retrospective Memory Questionnaire (PRMQ) scale was applied for retrospective memory assessment (19). The values of 58, 76, and 83 were used as the cutoff points for the ACE-R, respectively, for <4, 4–8, and > 8 schooling years (20, 21). Concerning the MMSE, we employed the cutoff points of 19 and 24, respectively, for 0 and up to 4 schooling years (22, 23). In addition, patients were defined as healthy if CDR = 0 and cognitively impaired if CDR = 0.5 (15). Functional impairment was defined by a score of 3 on the Pfeffer scale (24). Regarding psychiatric evaluation, we used a cutoff point of 3 on the GDS and 10 on the Beck inventory to diagnose depression (18, 25). In this study, cognitive impairment was defined when a cognitive complaint was confirmed by screening tests, regardless of functional impairment. Patients with cognitive complaints without objective impairment in the tests performed were characterized as subjective cognitive decline (SCD) (26).

### APOE genotyping analysis

According to the manufacturer's instructions, the patient's blood samples were collected in EDTA tubes, and subsequently, genomic DNA was extracted from peripheral blood leukocytes with the commercial PureLink™ Genomic DNA Mini Kit® (Invitrogen) (25). APOE genotypes were determined by real-time PCR (qPCR) using the TaqMan® allelic discrimination system (TaqMan® SNP Genotyping Assay, ThermoFisher®) (26). To this end, we used probes per sequences provided by the manufacturer: C___3084793_20 (rs429358) and C____904973_10 (rs7412), observing the information contained in the catalog number: 4351379, and similar protocols were used, described in the literature, for performing the technique. All analyses were performed in the QuantStudio® 5 qPCR platform (Applied Biosystems®, Foster City, CA, USA) (27).^2^^,^ ^3^^,^ ^4^^,^ ^5^

### Statistical analysis

Categorical data were expressed as absolute counts and percentages. Chi-square tests were used to evaluate the association among categorical data. Continuous data were first evaluated for normal distribution using the Kolmogorov–Smirnov test (28). Normal data were expressed as mean ± standard deviation (SD) and non-normal data as the median and interquartile range (IQR). Continuous data were compared among three groups per cognitive impairment (normal or cognitive unimpaired [CU] vs. cognitive impairment vs. subjective cognitive decline [SCD]). We compared normal data using one-way ANOVA with Tukey's *post hoc test*, and we adopted the Kruskal–Wallis test with Dunn's *post hoc test* for non-normal data (29). We analyzed data using SPSS software for Macintosh, version 23 (Armonk, NY: IBM Corp.). Values of *p* < 0.05 were considered statistically significant.

### Ethical aspects

The Research Ethics Committee of the Walter Cantídio University Hospital approved the study project under the number 4.092.933. All patients signed an Informed Consent Form with the right to privacy and confidentiality of the information obtained and could refuse to participate in the proposed activities and questions.

## Results

**Figure 1:**
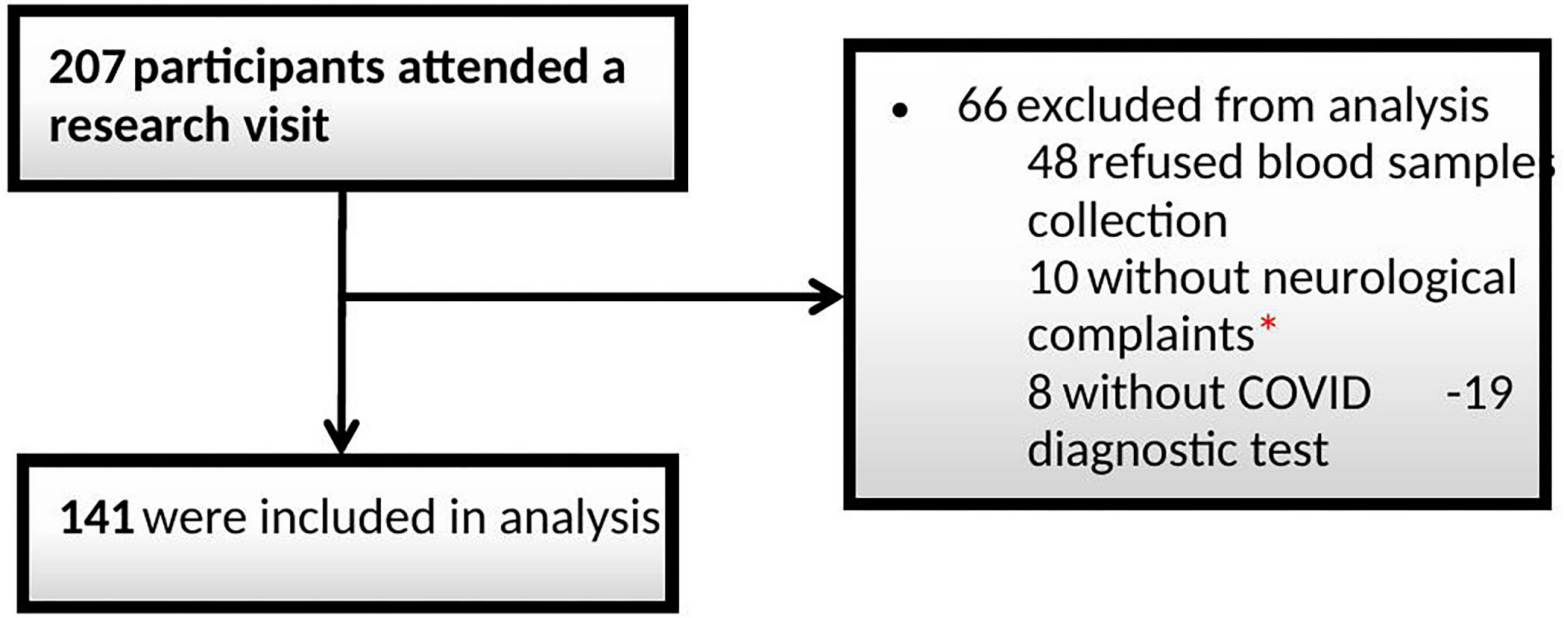
Flow diagram of participants. *E.g., headache, anosmia, cognitive complaints and others.

In total, 207 individuals were screened, of which 66 were excluded (48 for not having performed blood collection, 10 for not showing neurological symptoms, and 8 for not having tested positive for COVID-19 through tests) (Figure 1). The final number of patients included in this study was 141, and all the following analyses were conducted on them. Patients were evaluated, on average, 4.5 months after COVID-19.

**Figure 2:**
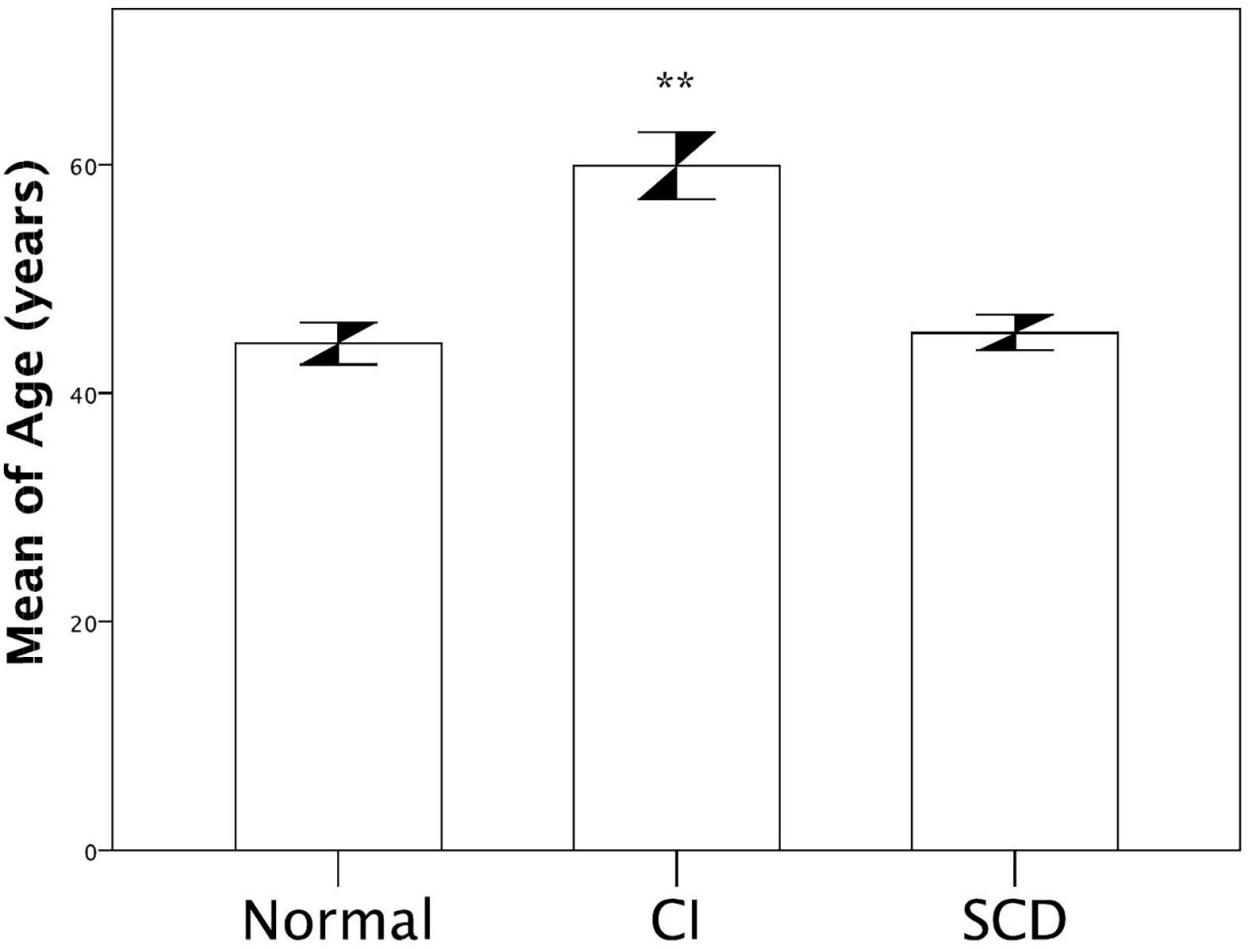
Comparison of age according cognitive status. ANOVA test was applied with Tukey post-test. ** *p* < 0.05 between CI vs. SCD and Normal in multiple using Tukey's test.

Table 1 presents a descriptive summary of the patients' characteristics. There was a female predominance (63.1%). The mean age was 48 years (16–90; SD = 14), with most patients having schooling >12 years (54.6%) (Figure 2). Most patients were not hospitalized in the acute phase of the disease (65.2%), and a minority had a severe clinical condition requiring admission to the intensive care unit (ICU) (3.5%). The most frequent APOE genotype was E3/E3 with 67.3% of cases, with a predominance of the E3 allele (96.5%); the second was the genotype E3/E4, corresponding to 23.4% of all cases, and the E4 allele (26.2%). In addition, 93 of the 141 patients (65.9%) had memory impairment symptoms as the main complaint. However, such complaint was objectively confirmed through screening tests in 25 patients (17.7%). In patients with cognitive impairment, we detected new dementia or deteriorated previous dementia in 2.8% of the total sample.

**Table 1:** Participant demographics, clinical characteristics, APOE genotype and cognition impairment.

**Variables**	**n**	**%**
**Gender**
Male	52	36.9%
Female	89	63.1%
**Scholarity (years)**
0	4	2.8%
1–4	6	4.3%
5–8	17	12.1%
9–12	37	26.2%
>12	77	54.6%
**Hospitalization**
No	92	65.2%
Yes	36	25.5%
**APOE genotype**		
E2/E2	1	0.7%
E2/E3	8	5.7%
E2/E4	1	0.7%
E3/E3	95	67.3%
E3/E4	33	23.4%
E4/E4	3	2.1%
**APOE allele**
E2	10	7.1%
E3	136	96.5%
E4	37	26.2%
**Cognition**
Normal	48	34,0%
CI	25	17.7%
SCD	68	48.2%

*CI, cognitive impaired; SCD, Subjective cognitive decline*.

**Figure 3:**
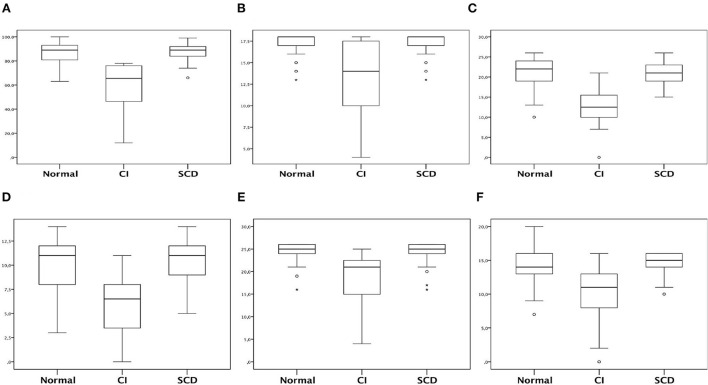
Box-plot representing total ACE-R and subitens scores comparison in relation of patients cognitive status. **(A)** Total ACE-R; **(B)** Attention and orientation; **(C)** Memory; **(D)** Fluency; **(E)** Language; **(F)** Visuospatial. *Kruskal-Wallis test was applied with Dunn post-test. **(A)** *p* < 0.05 between CI vs. SCD and Normal. CI, coginitve impaired; SCD, subjective cognitive decline.

Regarding ACE-R and its sub-items evaluation, the cognitive impairment group showed a worse profile in total ACE-R and all sub-items (Tables 2, 3). The cognitive impairment group had decreased total ACE-R and sub-items than the normal and SCD groups (*p* < 0.001) (Figure 3).

**Table 2:** Total ACER, ACER subitens, MMSE, PRMQ, Beck, GDS, Pfeffer, and MRC scores.

**Instruments**	**Mean**	**Minimum**	**Maximum**	**Standard deviation**	**Median**	**Mean (95%CI)**	
						**LL**	**UL**
Total ACE-R	82.5	12.0	100.0	15.1	87.0	80.0	85.1
Attention and orientation	16.5	4.0	18.0	2.6	18.0	16.1	17.0
Memory	19.4	0.0	26.0	5.1	20.0	18.6	20.3
Fluency	9.6	0.0	14.0	3.1	10.0	9.1	10.1
Language	23.5	4.0	26.0	4.3	25.0	22.7	24.2
Visuospatial	13.6	0.0	20.0	2.9	14.0	13.1	14.0
MMSE	27.4	10.0	30.0	3.7	29.0	26.8	28.0
PRMQ	7.0	5.0	25.0	4.0	5.0	6.3	7.7
Beck	5.2	0.0	21.0	6.6	0.0	4.0	6.4
GDS	3.7	0.0	12.0	3.6	3.0	2.1	5.3
Pfeffer	1.7	0.0	30.0	6.7	0.0	0.6	2.8
MRC before	0.0	0.0	2.0	0.2	0.0	0.0	0.1
MRC after	0.6	0.0	3.0	0.9	0.0	0.4	0.7

*LL, Lower limit; UL, Upper limit; ACE-R, Addenbrooke's Cognitive Examination-Revised; CDR, Clinical Dementia Rating; MMSE, Mini-Mental State Examination; MRC, Medical Research Council; GDS, Geriatric Depression Scale; PRMQ, Prospective and Retrospective Memory Questionnaire's scale*.

**Table 3:** Total ACE-R and subitens scores comparison in relation of patients cognitive status.

	**Cognitive status**			
	**Normal (*n* = 48)**	**CI (*n* = 25)**	**SCD (*n* = 68)**	*P*-value^*^
Total ACE-R	89 (81–93)	65.5 (46.5–76)	89 (84–92)	**<0.001** ^**A**^
Attention and	18 (17–18)	14 (10–17.5)	18 (17–18)	**<0.001** ^**A**^
orientation		
Memory	22 (19–24)	12.5 (10–15.5)	21 (19–23)	**<0.001** ^**A**^
Fluency	11 (8–12)	6.5 (3.5–8)	11 (9–12)	**<0.001** ^**A**^
Language	25 (24–26)	21 (15–22.5)	25 (24–26)	**<0.001** ^**A**^
Visuospatial	14 (13–16)	11 (8–13)	15 (14–16)	**<0.001** ^**A**^

*Continuous data expressed as median and interquartile range between parenthesis*.

* *Kruskal-Wallis test was applied with Dunn post-test. A: p <0.001 between CI vs. SCD, and p <0.001 between CI vs. Normal*.

*CI, cognitive impaired; SCD, Subjective cognitive decline*.

**Figure 4:**
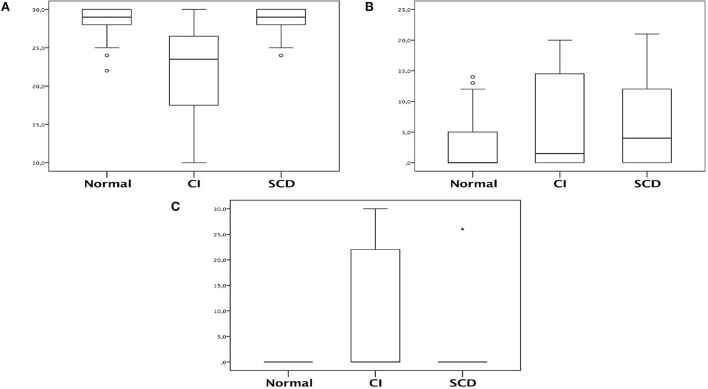
Box-plot representing MEEM, Beck and Pfeffer comparison in relation of patients cognitive status. **(A)** MMSE; **(B)** Beck; **(C)** Pfeffer. The asterisk symbol used to indicate the type of statistical test used to calculate *p*.

Furthermore, other tests showed alterations in the cognitive impairment group. The MEEM score decreased in the cognitive impairment group compared with the normal and SCD groups (median of 23.5 [IQR of 17.5–26.5] vs. 29 [28–30] vs. 29 [28–30], respectively, *p* < 0.001). Concerning Beck's depression inventory, a statistical difference was only observed between the SCD group and the normal group, where SCD had increased levels (*p* = 0.030). Regarding Pfeffer's score, cognitive impairment had increased levels compared with the normal and SCD groups (Table 4; Figure 4). Before and after, there was no statistical significance between the groups for evaluations with other scores, such as PRMQ, GDS, and MRC.

**Table 4:** MEEM, PRMQ, Beck, GDS, Pfeffer, and MRC scores comparison in relation of patients cognitive status.

	**Cognitive status**			
	**Normal (*n* = 48)**	**CI (*n* = 25)**	**SCD (*n* = 68)**	*P*-value^*^
MEEM	29 (28–30)	23.5 (17.5–26.5)	29 (28–30)	**<0.001** ^**A**^
PRMQ	5 (5–5.5)	5 (5–13)	5 (5–7)	0.079
Beck	0 (0–5)	1.5 (0–14.5)	4 (0–12)	0.030^**B**^
GDS	3 (0–4)	3 (2–11)	1.5 (0–6)	0.407
Pfeffer	0 (0–0)	0 (0–22)	0 (0–0)	**<0.001** ^**A**^
MRC before	0 (0–0)	0 (0–0)	0 (0–0)	0.885
MRC after	0 (0–1)	0 (0–1)	0 (0–1)	0.333

*Continuous data expressed as median and interquartile range between parenthesis. The bold values indicate the statistically significant signaled values*.

* *Kruskal-Wallis test was applied with Dunn post-test*.

*A: p <0.001 between CI vs. SCD and p <0.001 between CI vs. Normal*.

*B: p = 0.024 between Normal vs. SCD*.

*CI, cognitive impaired; SCD, Subjective cognitive decline*.

Table 5 shows the correlation between patients' cognitive impairment and other symptoms (anosmia, depression, and headache). The cognitive impairment group had a lower frequency of anosmia than the normal and SCD groups (4 vs. 40 vs. 34%, respectively, *p* = 0.005). Depression was more frequent in the SCD and cognitive impairment groups than in the normal group (50 vs. 40 vs. 27%, respectively, *p* = 0.046). A total of 48 patients developed depression after COVID-19. In total, nine patients were hospitalized in the acute phase of the disease. Moreover, they had a mean ACER score of 85.7 [62–99], 63% were women, and the mean age was 43.4 years.

**Table 5:** Comparison between cognitive impairment in relation to other symptoms.

	**Cognitive status**			
	**Normal = 48 *n* (%)**	**CI = 25 *n* (%)**	**SCD = 68 *n* (%)**	*P*-value^*^
**Anosmia**				**0.005**
No	29 (60.4)	24 (96)	45 (66.2)	
Yes	19 (39.6)	1 (4)	23 (33.8)	
**Depression**				**0.046**
No	35 (72.9)	15 (60)	34 (50)	
Yes	13 (27.1)	10 (40)	34 (50)	
**Headache**				0.291
No	37 (77.1)	19 (76)	44 (64.7)	
Yes	11 (22.9)	6 (24)	24 (35.3)	

*Categorical data expressed as absolute count and percentages between parenthesis. The bold values indicate the statistically significant signaled values*.

* *Chi-square test was used*.

*CI, cognitive impaired; SCD, Subjective cognitive decline*.

Table 6 shows patients' demographics and APOE genotyping with cognitive status correlation. Cognitive status had no association with APOE genotypes (*p* = 0.840) or alleles (Table 6). Conversely, the cognitive impairment was significantly more frequent in hospitalized patients and those with a lower education level (Table 6). Table 7 describes age comparisons concerning patients' cognitive status. The cognitive impairment group was significantly older than the SCD and normal groups (Table 7; Figure 2).

**Table 6:** Comparison between patients demographics and APOE genotype in relation to cognitive status.

	**Cognitive status**			
	**Normal = 48 *n* (%)**	**CI = 25 *n* (%)**	**SCD = 68 *n* (%)**	*P*-value^*^
**Gender**				0.104
Female	22 (45.8)	11 (44)	19 (27.9)	
Male	26 (54.2)	14 (56)	49 (72.1)	
**Scholarity (years)**				**<0.001**
Until 8 years	5 (10.4)	12 (48)	10 (14.7)	
9 years or more	43 (89.6)	13 (52)	58 (85.3)	
**Hospitalization**				**<0.001**
No	34 (79.1)	9 (36)	49 (81.7)	
Yes	9 (20.9)	16 (64)	11 (18.3)	
**APOE genotype**				0.840
E2/E2	0 (0)	0 (0)	1 (1.5)	
E2/E3	2 (4.2)	2 (8)	4 (5.9)	
E2/E4	0 (0)	0 (0)	1 (1.5)	
E3/E3	37 (77.1)	16 (64)	42 (61.8)	
E3/E4	8 (16.7)	6 (24)	19 (27.9)	
E4/E4	1 (2.1)	1 (4)	1 (1.5)	
**APOE allele**				
E2	2 (4.2)	2 (8)	6 (8.8)	0.618
E3	47 (97.9)	24 (96)	65 (95.6)	0.793
E4	9 (18.8)	7 (28)	21 (30.9)	0.335

*Categorical data expressed as absolute count and percentages between parenthesis. The bold values indicate the statistically significant signaled values*.

* *Chi-square test was used*.

*CI, cognitive impaired; SCD, Subjective cognitive decline*.

**Table 7:** Total sample age and according cognitive status.

	**Age (years)**				
	**Mean**	**Standard deviation**	**Minimum**	**Maximum**	*P*-value^*^
**Total sample**	48	14	16	90	-
**Cognitive status**					<0.001
Normal	44	13	17	69	
CI	60	15	23	90	
SCD	45	13	16	74	

* *ANOVA test was applied with Tukey post-test for multiple comparisons: p <0.001 between CI vs. SCD and p <0.001 between CI vs. Normal*.

## Discussion

Cognitive complaints are common during and after COVID-19, but few studies have objectively evaluated such complaints, especially after the acute phase of the disease (5). Furthermore, the literature has not yet reported the assessment of specific APOE haplotypes or genotypes with such cognitive complaints after COVID-19. In this study, we evaluated an outpatient population. Cognitive changes were the main complaints, even in mild cases with a low mean age of 48 years and an average assessment of 4 months after the COVID-19 diagnosis. Analyzed by a cognitive screening instrument, we confirmed objective cognitive deficits in some subjects. Furthermore, depression was more common in subjects with SCD compared to the normal group.

Other studies have evaluated the association between cognitive impairment and COVID-19, both in the acute/subacute phase (<12 weeks) or after this period (>12 weeks) of the disease (30, 31). This differentiation is crucial since other factors can contribute to cognitive complaints, such as hospitalization, hypoxemia, and delirium in the acute phase of the disease (32–34). Our study, for example, found a significantly higher number of subjects with cognitive complaints and cognitive impairment hospitalized during the acute phase of the disease. Possible explanations could be that hospitalized patients had more severe infection with a greater possibility of systemic complications, more significant inflammatory response, and prolonged hospitalization, all related to worse cognitive performance (33, 34). Negrini et al. evaluated cognitive impairment in discharged patients and demonstrated that cognitive malfunctioning appears to be linearly associated with the length of stay in the intensive care unit (35). Furthermore, two recent systematic reviews, one with meta-analysis, found a lower general cognition in patients with COVID-19 (36, 37). The meta-analysis with the Montreal Cognitive Assessment (MoCA) showed lower scores for patients with COVID-19 compared to healthy controls (37). Conversely, other factors may account for the symptoms after the acute phase, such as inflammatory markers (4). This last finding is important, as previous evidence shows a possible causal role of microglial inflammation and Alzheimer's disease (38).

To correlate a possible genetical predisposition and a significant risk of developing cognitive impairment, we performed an APOE genotyping for SNPs rs429358 and rs7412 in those patients, which are widely discussed in the literature as responsible for increasing the risk of dementia and cognitive impairment (39–42). APOEs play a vital role in lipid transport and metabolism, thus influencing the risk of cardiovascular disease (10). They also have neuroprotective functions, including the E4 haplotype associated with an increased risk of Alzheimer's disease (9). The E3 allele, in turn, does not display a greater or lesser risk of developing Alzheimer's disease. Most of our sample had E3/E3 genotype (67.3%), and the E4 allele was detected in 26.2% of the cases, similar to previous studies in the Brazilian population where the E3/E3 genotype was predominant (43). Our study did not evidence significant difference between groups regarding genotypes found or specific alleles, perhaps due to a low number of participants, mainly in the cognitive impairment group, despite a trend of a direct correlation between the cognitive impairment/SCD groups and the E4 allele, the same implicated in an increased risk of sporadic Alzheimer's disease (9). However, other factors could trigger cognitive impairment and do not necessarily involve specific APOE polymorphisms, such as inflammation, cerebral ischemia, and hypoxemia. To the best of our knowledge, no study has evaluated this association of post-COVID cognitive impairment with APOE polymorphism to date.

After the acute disease phase of COVID-19 infection, some patients have described some persistent symptoms, such as memory complaints, receiving the name “long-haulers” by some authors (44). This clinical picture is similar to that of myalgic encephalomyelitis/chronic fatigue syndrome and the symptoms described after influenza (45, 46). However, post-COVID-19 symptoms occur at a higher frequency than influenza (46).

In this study, memory complaints without objective evidence were common and found in 65.9% of patients. Similarly, objective cognitive impairment detected through screening tests occurred in 17.7% of patients. These two backdrops refer to subjective cognitive decline and mild cognitive impairment conditions, whose gold standard for the diagnosis lies in extensive neuropsychological assessment not performed in these patients (26, 47). These conditions are essential given the possible progression to Alzheimer's disease (48, 49).

Psychiatric disorders may also be responsible for cognitive complaints (50). In our study, subjects with SCD had significantly higher scores on Beck's depression inventory than the normal group, which is relevant since patients with SCD had more depressive symptoms (26). Furthermore, Ismael et al. evaluated patients with mild COVID-19 and showed that 26.2% of patients had depressive symptoms 2 months after infection (51). Moreover, the impact on patients' lives also contributes to depressive symptoms (52).

In addition, our study found an inverse correlation between cognitive impairment and anosmia, which was in disagreement with other studies. Cristillo et al. found a direct association between cognitive impairment and olfactory dysfunction in patients after COVID-19 but in an old sample, making it possible as a brain aging marker (53). Previous studies demonstrated that olfactory dysfunction occurred in elderly patients along with cognitive impairment as brain aging markers (54). Finally, our study did not find associations between cognitive impairment and headache. Notwithstanding this, this association between headache and cognitive impairment can be found in patients after the acute phase of COVID-19 (55).

The most affected cognitive impairment group domain in ACE-R sub-items was the memory, as found in other studies (56, 57), which is relevant because limbic structures may suffer from inflammation (58). Hosp et al. evaluated brain PET-FDG in patients with acute phase COVID-19 and showed limbic involvement besides other brain structures (30). There was also a worse performance in the other ACE-R sub-items of attention, fluency, language, and visuospatial functions, but it was lighter than the memory sub-item.

Our study has some significant limitations. First, there was no control group. Additionally, our study has a selection bias, as we selected patients with neurological symptoms. We also did not perform a broader neuropsychological assessment to determine which cognitive domains were more affected and objectively assess other patients with subjective memory complaints without objective evidence in screening tests. Furthermore, a neuropsychological assessment is part of the diagnostic criteria for cognitive impairment and SCD; as it was not performed, the diagnosis of these conditions was impaired.

Moreover, despite differences found between cognitive impairment and normal/SCD groups' ACE-R scores, we should mention that there were essential differences between these groups regarding age and schooling, respectively, lower and higher in the normal/SCD groups, which may explain these differences found in ACE-R. Furthermore, selecting patients whose symptoms persisted for more than 3 months created a noteworthy bias since those whose symptoms disappeared before this period did not seek care. Furthermore, as the number of dementia cases found was low, we did not adjust for the total sample, and this study may achieve only a moderate effect if it exists since the total sample size should be *n* = 1,283 to achieve a small effect (w = 0.3) with a power of 80% for an association between APOE status and cognition on independence tests (59). Finally, there was no neuroimaging evaluation, precluding analysis of associations between complaints and radiological correlations. Nonetheless, this study is the most extensive series of patients so far, emphasizing cognitive complaints in an outpatient setting after the disease's acute phase. Furthermore, our sample consisted of patients with mild forms of the disease and after the acute and subacute phases of the disease, allowing us to show the persistent symptoms even in this population. Finally, APOE polymorphism analysis and possible associations with other symptoms strengthen our study.

In conclusion, our study helps to build knowledge about patients with post-COVID-19 cognitive manifestations. Our study reveals that cognitive complaints are common in patients with COVID-19, even after the acute disease phase and in mild cases, similar to other studies in the literature (36, 37). Hospitalized participants may have a higher risk of cognitive impairment. Moreover, APOE genotypes or haplotypes may not significantly play a role in the COVID-19 cognitive impairment. Longitudinal follow-up of these patients is critical to determine whether this cognitive impairment persists after a certain period. Furthermore, a neuropsychological assessment of these patients is crucial for better characterization of SCD or MCI and determining the most affected cognitive domains. Finally, it would be necessary for those with cognitive impairment to evaluate biomarkers of neurodegenerative diseases in cerebrospinal fluid or plasma, such as amyloid Beta 1–42, phosphorylated tau, and light chain neurofilament, thus bringing a link between COVID-19 and the onset or worsening of neurodegenerative diseases (60, 61).

## Data availability statement

The raw data supporting the conclusions of this article will be made available by the authors, without undue reservation.

## Ethics statement

The studies involving human participants were reviewed and approved by Research Ethics Committee of the Walter Cantídio University Hospital under the number 4.092.933. The patients/participants provided their written informed consent to participate in this study.

## Author contributions

Conception and design of work: JT-J, MS-N, and PB-N. Acquisition, analysis, or interpretation of data and work and drafting the work: JT-J and PB-N. All authors were involved in the critical review of the manuscript for important intellectual content.

## Funding

The authors are grateful to the Brazilian National Council for Scientific and Technological Development (CNPq) for the funding of the Fellowship to authors PB-N and RM; to the Coordination for the Improvement of Higher Education Personnel—Brazil (CAPES) for the funding to authors PB-N and RM (88881.505364/2020-01); and to the Ceará Foundation to Support Scientific and Technological Development (FUNCAP) for the funding to author CG.

## Conflict of interest

The authors declare that the research was conducted in the absence of any commercial or financial relationships that could be construed as a potential conflict of interest.

## Publisher's note

All claims expressed in this article are solely those of the authors and do not necessarily represent those of their affiliated organizations, or those of the publisher, the editors and the reviewers. Any product that may be evaluated in this article, or claim that may be made by its manufacturer, is not guaranteed or endorsed by the publisher.

## Footnotes

^1^https://www.who.int/emergencies/diseases/novelcoronavirus-2019 (accessed August 17, 2021).

^2^ https://www.thermofisher.com/order/catalog/product/K182002#/K182002

^3^ https://www.thermofisher.com/order/catalog/product/4351379#/4351379

^4^ https://www.thermofisher.com/order/genome-database/details/genotyping/C___3084793_20?CID=&ICID=&subtype=

^5^ https://www.thermofisher.com/order/genome-database/details/genotyping/C____904973_10?CID=&ICID=&subtype=
